# Serum levels of B-cell activating factor of the TNF family (BAFF) correlate with anti-Jo-1 autoantibodies levels and disease activity in patients with anti-Jo-1positive polymyositis and dermatomyositis

**DOI:** 10.1186/s13075-018-1650-8

**Published:** 2018-07-27

**Authors:** Olga Kryštůfková, Hana Hulejová, Heřman F. Mann, Ondřej Pecha, Ivana Půtová, Louise Ekholm, Ingrid E. Lundberg, Jiří Vencovský

**Affiliations:** 1 Institute of Rheumatology, Prague, Czech Republic; 2 Department of Rheumatology, First Faculty of Medicine, Charles University, Prague, Czech Republic; 3 Technology Centre ASCR, Prague, Czech Republic; 4 Division of Rheumatology, Department of Medicine, Karolinska University Hospital, Solna, Karolinska Institutet, Stockholm, Sweden

**Keywords:** BAFF, Anti-Jo-1 autoantibodies, ILD, Myositis

## Abstract

**Background:**

B-cell activating factor of the tumour necrosis factor family (BAFF) plays a role in autoantibody production and is elevated in dermatomyositis (DM) and anti-Jo-1-positive polymyositis (PM). We investigated the inter-relationships between serum levels of BAFF, anti-Jo-1 autoantibodies, and disease activity.

**Methods:**

Serum levels of BAFF and anti-Jo-1 antibodies measured by enzyme-linked immunosorbent assay (ELISA) were compared to levels of myoglobin, creatine kinase (CK), aminotransferases (alanine (ALT) and aspartate (AST)), C-reactive protein (CRP), and disease activity assessed by the Myositis Disease Activity Assessment Tool in 63 anti-Jo-1 antibody-positive DM/PM patients. Serial serum samples collected at 2 (46 cases) and 3–5 time points (23 cases) were included. Relationships between BAFF, anti-Jo-1, disease activity, CRP, and their longitudinal changes were evaluated using correlation analysis, multiple regression (MR), path analysis (PA), and hierarchical linear models (HLM).

**Results:**

Cross-sectional assessment demonstrated significant correlations between the levels of BAFF and anti-Jo-1 antibodies which were associated with levels of CK, myoglobin, AST, and CRP, as well as multivariate associations between BAFF, anti-Jo-1 antibodies, and CK levels. PA revealed direct effects of anti-Jo-1 antibodies on CK (β = 0.41) and both direct (β = 0.42) and indirect (through anti-Jo-1 antibodies; β = 0.17) effects of BAFF on CK. Changes in levels of both BAFF and anti-Jo-1 between two time points (Δ) were associated with Δmyoglobin and Δaminotransferases and changes of BAFF correlated with ΔCK, Δcutaneous, Δmuscle, Δglobal, and Δskeletal disease activities.

The longitudinal analysis showed a high intra-individual variability of serum levels of BAFF over time (97%) which could predict 79% of the variance in anti-Jo-1 levels. The anti-Jo-1 variability was explained by inter-individual differences (68%). The close longitudinal relationship between levels of BAFF, anti-Jo-1, and disease activity was supported by high proportions of their variance explained with serum levels of CK and CRP or pulmonary and muscle activities.

**Conclusion:**

Our findings of associations between levels of BAFF and anti-Jo-1 antibodies in serum and myositis activity suggest a role of this cytokine in disease-specific autoantibody production as part of disease mechanisms, and support BAFF as a potential target for intervention in anti-Jo-1-positive myositis patients.

**Electronic supplementary material:**

The online version of this article (10.1186/s13075-018-1650-8) contains supplementary material, which is available to authorized users.

## Background

Polymyositis (PM) and dermatomyositis (DM) are chronic, inflammatory disorders characterised by muscle weakness and by the presence of inflammatory infiltrates in the skeletal muscle [1]. Other organs such as the skin and lungs are frequently involved. Myositis-specific antibodies (MSA) or myositis-associated antibodies are present in up to 80% of PM/DM patients [2]. The anti-histidyl-tRNA synthetase (anti-Jo-1) autoantibodies are the most frequent MSA (present in 20–30% of DM/PM patients) [3] and are associated with a distinct clinical phenotype (i.e. anti-synthetase syndrome), characterised by myositis, Raynaud’s phenomenon, interstitial lung disease (ILD), arthritis, and skin changes of the hands [4]. The observation that anti-Jo-1 antibodies could be present before the onset of clinical symptoms may suggest a possible role of the antibody in the pathogenesis of this subset of myositis [5, 6].

B-cell activating factor of the tumour necrosis factor family (BAFF; also known as B lymphocyte stimulator, or BLyS) is crucial for B-cell maturation and survival. BAFF is also believed to play a role in autoantibody production [7]. High serum levels of BAFF have been reported in patients with autoimmune diseases [8–15] and are associated with disease activity and the presence or levels of autoantibodies [9, 10, 12–14, 16]. In patients with myositis, elevated serum levels of BAFF were found to correlate with serum levels of creatine kinase (CK). In addition, the linear regression analysis of variance (ANOVA) model confirmed that anti-Jo-1 antibodies and ILD are the main influencing factors for levels of BAFF, particularly in PM patients [17]. Based on this observation and on the previously reported correlations between serum levels of anti-Jo-1 antibodies and disease activity [18], we aimed to study associations between BAFF and anti-Jo-1 antibody levels in longitudinally collected serum samples and their relation to standardised clinical measures and laboratory markers of disease activity in patients with DM/PM, with a focus on early cases and the subgroup defined by the presence of ILD. C-reactive protein (CRP) is currently not used as a biomarker of disease activity in myositis. However, correlations between serum levels of CRP and lung function tests among anti-Jo-1-positive myositis patients with ILD have been reported [19], and elevated CRP levels have been described as a risk factor for developing ILD in patients with myositis [20, 21]. Moreover, an association between increased BAFF and CRP levels in the serum was reported in patients with systemic lupus erythematosus (SLE) [13, 22]. Therefore, we also included analysis of associations with serum levels of CRP.

## Methods

### Patients and controls

All patients with myositis who had tested positive for anti-Jo-1 antibodies over a period of 9 years in the Institute of Rheumatology Prague, Czech Republic (*n* = 53), and ten patients from the Rheumatology Clinic at Karolinska University Hospital, Stockholm, Sweden, with available longitudinally collected clinical data and paired serum samples were included in the study. Of these, 41 patients were diagnosed as probable or definite PM and 22 as DM based on the Bohan and Peter criteria [23, 24] and 48 of them (76%) had myositis-associated ILD defined by pathological chest x-ray or computed tomography (CT), and abnormal pulmonary function tests.

Clinical data and serum samples were collected prospectively at consecutive clinical visits over 9 years using a disease register in both hospitals. The prospectively collected disease activity assessments were available in 58 patients (20 DM and 38 PM) in the form of the Myositis Disease Activity Assessment Tool (MDAAT) according to the International Myositis Assessment and Clinical Studies (IMACS) Group, including extramuscular, muscular, and physician’s global score of disease activity by visual analogue scale (VAS) and MYOACT score [25]. The Swedish and Czech cohorts had comparable demographic and clinical characteristics, but Swedish patients were older and had longer disease duration (data not shown). Sera from 41 age- (mean 51.1 ± 11.7 years) and gender (female:male = 28:13)-comparable Czech healthy individuals without any known inflammatory disease or recent infection were used as controls. Ethics committees of both institutions approved the study and informed consent was obtained from the participants.

Altogether, 143 serum samples were collected within the study. The earliest available serum sample obtained from each patient was used for the cross-sectional analysis. Paired samples obtained during regular clinic appointments (median 1.5 years, range 0.4 – 7.2) with clinical activity assessment by MDAAT at both visits were available in 46 cases (36 Czech and 10 Swedish, 17 DM and 29 PM patients). Sera from three to five time points collected from 23 patients were included to analyse longitudinal associations between BAFF and anti-Jo-1 antibody levels and their relation with disease activity. Intervals between the first and last sample ranged from 0.6 to 8.9 years and the time flow of individual visits is represented in Additional file 1: Figure S1.

### Laboratory measurements

Serum levels of CK, myoglobin, alanine aminotransferase (ALT), aspartate aminotransferase (AST), and CRP were routinely measured in local laboratories with comparable reference levels for Czech and Swedish cohorts (Table 1). All other laboratory analyses were performed in the same laboratory at the Institute of Rheumatology in Prague in serum samples stored at −80 °C until analysis.

**Table 1:** Demographic characteristics, clinical, and laboratory data of patients at time of blood sampling at initial evaluation

	DM (*n* = 22)		PM (*n* = 41)		All (*n* = 63)	
Female:male	15:7		28:13		43:20	
Age (years), mean ± SD	52.5 ± 10.9		52 ± 12.6		52.2 ± 11.9	
Years of symptoms	5.9 (0.1–31.0)		3.8 (0.2–23.8)		4.1 (0.1–31.0)	
Years from diagnosis	1.3 (0–29.0)		0.8 (0–23.3)		1.0 (0–29.0)	
Early cases^a^, *n* (%)	10 (45%)		20 (49%)		30 (48%)	
ILD, *n* (%)	15 (68%)		33 (80.5%)		48 (76%)	
Medication, *n* (%)						
GC	20 (91%)		36 (88%)		56 (89%)	
DMARDs	11 (50%)		23 (56%)		34 (54%)	
No therapy	1 (4.5%)		4 (10%)		5 (8%)	
Dose of GC (mg/day)^b^	15 (0–80)		17.5 (0–85)		17.5 (0–85)	
MDAAT visual analogue scale (mm)	(*n* = 20)		(*n* = 38)		(*n* = 58)	
Constitutional	1 (0–35)		0 (0–25)		0 (0–35)	
Cutaneous	10 (0–30)		0 (0–21)		1 (0–30)	
Skeletal	0 (0–43)		0 (0–63)		0 (0–63)	
Gastrointestinal	0 (0–17)		0 (0–27)		0 (0–27)	
Pulmonary	11 (0–86)		13 (0–66)		12.5 (0–86)	
Cardiac	0 (0–10)		0 (0–36)		0 (0–36)	
Other	0 (0–17)		0 (0–29)		0 (0–29)	
Extramuscular global	11.5 (0–64)		17.5 (0–44)		15 (0–64)	
Muscle	7.5 (0–82)		11.3 (0–71)		11 (0–82)	
Global	21 (0–79)		16.5 (0–59)		17.5 (0–79)	
MYOACT score^c^	0.07 (0–0.26)		0.05 (0–0.23)		0.06 (0–0.26)	
	*n*		*n*		*n*	
BAFF (ng/ml)	22	2.1 (0.8–20.9)	41	1.7 (0.3–18.7)	63	1.8 (0.3–20.9)
anti-Jo-1 (kU/l)	22	127 (0.6–2135)	41	178 (0.8–3605)	63	162 (0.6–3605)
CK (μkat/l)	22	1.8 (0.25–94.5)	41	3.6 (0.3–78.4)	63	2.6 (0.25–94.5)
Myoglobin (μg/l)	19	80 (24 - 5313)	34	115 (10.6–3498)	53	94 (10.6–5313)
ALT (μkat/l)	22	0.4 (0.1–6.7)	40	0.5 (0.1–3.6)	62	0.5 (0.1–6.7)
AST (μkat/l)	21	0.5 (0.1–5.1)	40	0.4 (0.2–6.5)	61	0.4 (0.1–6.5)
CRP (mg/l)	22	3.0 (0.2–29.4)	38	3.5 (0.5–54.8)	60	3.25 (0.2–54.8)

Data are shown as median (range; minimum–maximum) unless otherwise stated

Myoglobin normal levels < 92 μg/l for men and < 76 μg/l for women

*ALT* alanine aminotransferase (normal levels < 0.75 μkat/l for men and < 0.57 μkat/l for women), *AST* aspartate aminotransferase (normal levels < 0.58 μkat/L for men and < 0.52 μkat/L for women), *BAFF* B-cell activating factor of the tumour necrosis factor family, *CK* creatine kinase (normal levels for Swedish cohort < 2.5 μkat/L for men and < 2.0 μkat/L for women, and for Czech cohort < 2.85 μkat/l and < 2.42 μkat/l), *CRP*= C-reactive protein (normal levels < 5 mg/l), *DM* dermatomyositis, *DMARD* disease-modifying anti-rheumatic drug, *GC* glucocorticoids, *ILD* interstitial lung disease (ever present), *MDAAT* Myositis Disease Activity Assessment Tool, *PM* polymyositis

^a^Early case = disease duration up to 6 months

^b^Equivalent of prednisone

^c^MYOACT score is the sum of the 10-cm visual analogue scale scores for each of the six individual organ systems divided by the total maximum possible score

The levels of BAFF were measured in serum samples by an enzyme-linked immunosorbent assay (ELISA) according to the manufacturer’s instructions (R&D Systems, Inc., Minneapolis, MN, USA). The cut-off was defined as the mean plus two standard deviations of the control subjects.

Anti-Jo-1 levels were measured in the same patient’s serum samples with the use of ELISA according to the manufacturer’s instructions (Orgentec, Mainz, Germany). The declared cut-off range for positivity was < 15 kU/l with borderline values 15–25 kU/l. To confirm anti-Jo-1 positivity, patient samples were tested using myositis Western blot (Anti-Myositis–Antigen EUROLINE-WB, Euroimmun, Lubeck, Germany) and line blot assay (Myositis-LIA, IMTEC, Berlin, Germany).

The ELISA test revealed levels of anti-Jo-1 antibodies above the upper detection limit of the assay in six out of 143 serum samples that were not available for further titration; therefore, they were excluded from parametric statistical analyses. Six patients had initial levels of anti-Jo-1 antibodies below the cut-off 15 kU/l, but samples were found to be confirmed positive using line blot and/or Western blot assays.

### Statistical analyses

Statistical analyses were performed using GraphPad Prism 5 (GraphPad Software, Inc., San Diego, CA, USA) and SPSS 17.0 (SPSS, Inc., Chicago, IL, USA). With respect to non-normal distribution of data, non-parametric tests were used or the logarithmical transformation to normality was applied where appropriate. For analysis of differences between groups, Mann-Whitney *U* test and for comparison of changes between two time-points Wilcoxon’s signed rank test were performed. Spearman’s rank order test (correlation coefficient rho abbreviated here as *r*) was used for correlations of parameters in cross-sectional evaluation and changes between two time points (Δ; computed by subtraction of values at the second visit from values obtained at first visit). Contingency tables were evaluated with Fisher’s exact test. The *p* values below 0.05 were considered as statistically significant. The Bonferroni correction of alpha values was introduced where appropriate. Based on the distribution, data are presented as median (range; minimum–maximum) values or (for age) as mean ± SD.

### Multivariate analysis

Multiple regression (MR) of serum levels of BAFF, anti-Jo-1 antibodies, and CRP (as independent variables), and CK (as dependent variable) was performed using SPSS package 17.0 (SPSS, Inc., Chicago, Illinois, USA). The path analysis (PA) of these variables was performed with use of the software package LISREL 8.8 (Scientific Software International, Inc., Lincolnwood, IL, USA) [26] on a covariance matrix derived from the logarithmically transformed variables with function ln(1 + *x*), due to their positively skewed distributions [27]. The maximum likelihood (ML) method was selected to estimate correct standard errors and the associated goodness-of-fit statistics [28, 29]. Models and their variants were evaluated by several fit indices: the chi-square statistics, root mean square error of approximation (RMSEA), comparative fit index (CFI), and parsimony normed fit index (PNFI). RMSEA < 0.05 and CFI > 0.95 were considered as acceptable model fit. Lower values of chi-square statistics and higher values of PNFI also indicate a good fit. Only significant standardised path coefficients (β) were accepted.

### Analysis of longitudinal data

None of the traditional ANOVA-like models is appropriate for the analysis of repeated measures over time of the unbalanced design of longitudinal collection of samples (as illustrated in Additional file 1: Figure S1). Therefore, the two-level hierarchical linear model (HLM) was designed separately for BAFF, anti-Jo-1, and CK using the HLM 6 package [30]. The principles of HLM are explained in detail in Additional file 2 [31].

A sequence of three consecutive models was accomplished. Firstly, the unconditional model was used for evaluation of the variance components situated within patients (lower level 1; 3 and more time points) and between patients (higher level 2; 23 cases) [32]. Their ratio, the intra-class correlation coefficient (ICC), described the proportion of the total variance between patients. Next, the unconditional growth model with time as the independent variable was applied as an indicator of intensity of associations over time. Finally, the model with one time-varying independent variable was used to assess the percentage of the variance of either BAFF, anti-Jo-1, or CK explained by a relevant variable measured repeatedly over time along with a given dependent variable (pseudo *R*^*2*^).

Since the limited sample size of longitudinally collected samples entered the HLM, only few variables representing the hypothesis were included to keep it in proportion with the sample size and to let the path-analytic model and HLM to be identified.

## Results

### Patient group characteristics

Patient demographic, laboratory, and clinical characteristics at initial assessment are summarised in Table 1. DM and PM cohorts had comparable disease activity (except for cutaneous VAS), descriptive characteristics, and frequency of treatment with glucocorticoids (GC) or disease-modifying anti-rheumatic drugs (DMARDs), with comparable daily doses of GC. Patients with ILD had higher pulmonary activity, extramuscular global assessment and global disease activity VAS, and MYOACT score compared with non-ILD patients (median values in ILD: 20, 19, 19 mm and 0.75 index; non-ILD: 0, 4, 6.5 mm and 0.27 index; *p* < 0.05 for all) but otherwise comparable disease activity, laboratory parameters, and clinical characteristics. A shorter duration of symptoms (*p* = 0.04) and time from diagnosis (*p* = 0.006) at inclusion was seen in patients with ILD.

Early cases (up to 6 months after diagnosis of myositis) constituted 48% of the total cohort (*n* = 30). These had higher disease activity as assessed by MYOACT score (median 0.87 vs 0.31), extramuscular global assessment, global, muscle and pulmonary activity VAS or serum levels of ALT (23.0, 25.0, 16.5, 23.0 mm and 0.57 μkat/l) when compared with patients with longer disease duration (7.5, 8.0, 6.5, 6.5 mm, 0.35 μkat/l; *p* < 0.05 for all after Bonferroni correction).

### Serum levels of BAFF and anti-Jo-1 antibodies and their mutual correlation at initial evaluation

Patients with PM/DM had higher serum levels of BAFF (median 1.8, range 0.3–20.9 ng/ml) compared with healthy individuals (0.8, 0.4–2.0 ng/ml, *p* < 0.0001), and elevated levels were documented in 54% of patients.

The serum levels of anti-Jo-1 antibodies had a high variability between individual patients with a wide range (Table 1) and the distribution was skewed towards lower values.

Serum levels of both BAFF and anti-Jo-1 were comparable between subgroups of patients with DM and PM (Table 1; BAFF, *p =* 0.30; anti-Jo-1, *p* = 0.13), and with or without ILD (median and range of BAFF: ILD = 1.8, 0.5–20.9, and non-ILD = 1.5, 0.3–8.6 ng/ml; *p* = 0.56; or anti-Jo-1: ILD = 173.6, 0.8–3604, and non-ILD = 151.8, 0.55–2063 kU/l; *p* = 0.36).

Positive correlations were found in the whole patient cohort between serum levels of BAFF and anti-Jo-1 antibodies (*r* = 0.42, *p* = 0.0006, *n* = 63), as well as in subgroups with DM or PM and with or without ILD (Table 2).

**Table 2:** Correlations between serum levels of BAFF and anti-Jo-1 antibodies with serum levels of muscle enzymes (CK and AST), myoglobin, and CRP at baseline evaluation in myositis patients and in subgroups with dermatomyositis/polymyositis, with or without lung involvement, and in patients with short disease duration

		anti-Jo-1		CK		Myoglobin		AST		CRP	
		*r*	*p*	*r*	*p*	*r*	*p*	*r*	*p*	*r*	*p*
All (*n* = 63)	BAFF	0.42	0.0006**	0.50	< 0.0001***	0.39	0.004*	0.45	0.0003**	0.44	0.0004**
	anti-Jo-1	–	–	0.47	0.0001***	0.46	0.0006**	0.28	0.03	0.38	0.003*
PM (*n* = 41)	BAFF	0.43	0.005*	0.63	< 0.0001***	0.42	0.01	0.43	0.006*	0.48	0.002*
	anti-Jo-1	–	–	0.44	0.004*	0.46	0.006*	0.18	0.26	0.41	0.01
ILD (*n* = 48)	BAFF	0.38	0.008	0.49	< 0.0001***	0.40	0.01	0.46	0.001*	0.44	0.003*
	anti-Jo-1	–	–	0.49	< 0.0001***	0.58	< 0.0001***	0.30	0.04	0.47	0.001*
Early (*n* = 30)	BAFF	0.57	0.001*	0.63	0.0002**	0.55	0.0025*	0.61	0.0003**	0.41	0.03
	anti-Jo-1	–	–	0.69	< 0.0001***	0.74	< 0.0001***	0.56	0.001*	0.34	0.06

*AST* aspartate aminotransferase, *BAFF* B-cell activating factor of the tumour necrosis factor family, *CK* creatine kinase, *CRP* C-reactive protein, *Early* early case, defined as duration up to 6 months from diagnosis, *ILD* interstitial lung disease, *PM* polymyositis

*p* = *p* value; *alfa = 0.05 (0.006 after Bonferroni correction); **alfa = 0.01 (0.001 after Bonferroni correction); ***alfa = 0.001 (0.0001 after Bonferroni correction)

### Associations of serum levels of BAFF and anti-Jo-1 antibodies with laboratory and clinical parameters of disease activity and CRP at initial evaluation

Serum levels of BAFF or anti-Jo-1 were positively associated with serum markers of muscle involvement (levels of CK, myoglobin, and AST; Table 2 and Additional file 3), particularly in patients with early disease.

When analysing the disease activity measures, a positive correlation was found between levels of BAFF and cutaneous activity (Spearman’s correlation coefficient *r* = 0.46, *p* = 0.04, *n* = 20) in patients with DM.

A significant correlation of serum levels of CRP with levels of BAFF (*p* = 0.0004) and anti-Jo-1 antibodies (*p* = 0.003; Table 2) was recorded. Therefore, we incorporated associations of CRP into further analyses. The baseline levels of CRP in the serum were significantly higher in patients compared with healthy individuals (median 3.25 mg/l, range 0.2–54.8 mg/l vs 1.3 mg/l, 0.4–3.5 mg/l; *p* < 0.001). Elevation of CRP levels above the reference limit (5 mg/l) occurred in 38% of the entire patient group and in 47% of patients with early disease. CRP levels were associated with muscle involvement (serum levels of CK (*r* = 0.30; *p* = 0.02, *n* = 60) and myoglobin (*r* = 0.42, *p* = 0.002, n = 60)0 as analysed in the whole group of patients and in subsets with PM or with ILD (CK: *r* = 0.42 or 0.30, *p* = 0.009 or 0.045, *n* = 38 or 45; myoglobin: *r* = 0.53 or 0.40; *p* = 0.008 or 0.01, *n* = 33 or 40, respectively). No correlation was found between CRP levels and clinical assessments of disease activity, including constitutional and skeletal VAS.

### Multivariate analysis of the cross-sectional relationships between serum levels of BAFF, anti-Jo-1 antibodies, CK, and CRP

The associations among bivariate correlated variables were analysed with multiple regression in the entire cohort of patients (*n* = 63). Serum levels of BAFF, anti-Jo-1 antibodies, and CRP were included as independent variables, and serum levels of CK as a dependent variable. This analysis showed significant associations both for serum levels of CK and BAFF (with path coefficient β = 0.38; *p* = 0.005) and levels of CK and anti-Jo-1 antibodies (β = 0.28; *p* = 0.02).

**Fig. 1:**
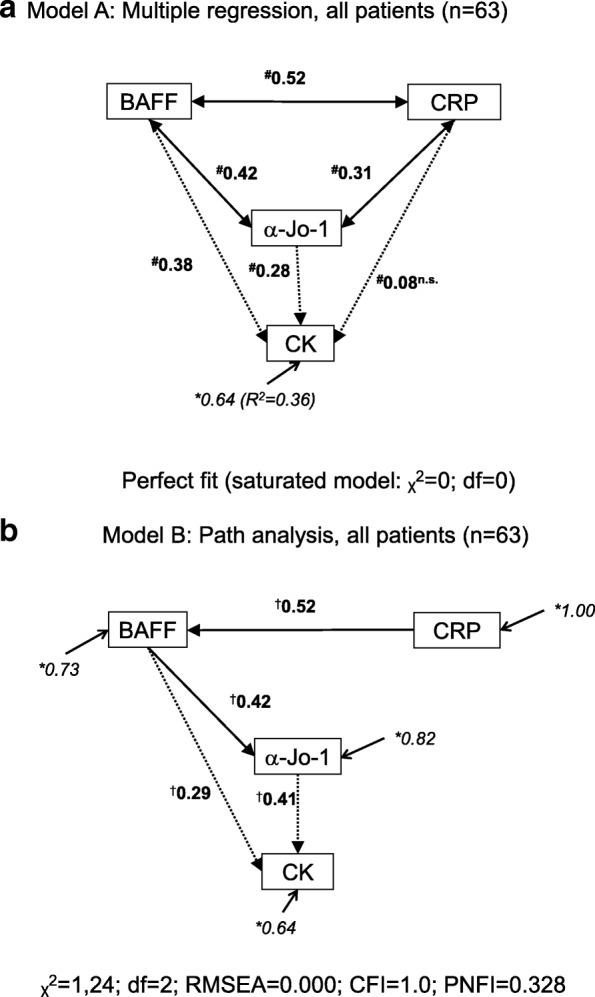
Associations between baseline serum levels of B-cell activating factor of the tumour necrosis factor family (BAFF), anti-Jo-1 antibodies (α-Jo-1), and C-reactive peptide (CRP) (as independent variables), and creatine kinase (CK) (as dependent variable) estimated by multivariable analysis in LISREL software [26]. **a** Multiple linear regression (MR) with standardised regression (path) coefficients (β)^#^. The mutual associations between independent variables (BAFF, anti-Jo-1, and CRP serum levels), found by MR, are depicted with two headed arrows and their influence on the dependent variable (CK) by one headed arrows. The non-significant path coefficient (*p* = 0.53) is marked as n.s. **b** Path analysis (PA) with path coefficients (β)^†^ and residual variances (Θ)* for all patients. One-headed arrows in PA represent significant direct effects, suggesting possible causal relationship. Only significant standardised path coefficients (*p* < 0.05) are shown. The best fit parameters of PA are shown below the diagrams: chi-square statistics (χ^2^), root mean square error of approximation (RMSEA), comparative fit index (CFI), and parsimony normed fit index (PNFI)

The possible causal relationships among these variables were further evaluated with the path analysis (PA) as shown by the path diagram (Fig. 1a). The modification of the MR model (explained in detail in Additional file 4) resulted in the PA model (Fig. 1b) where the dependence of anti-Jo-1 levels on BAFF levels (β = 0.42) appeared. Also, the positive effect of serum levels of anti-Jo-1 antibodies (β = 0.41) and BAFF on serum CK levels was recorded. The effect of BAFF on CK was both direct (β = 0.29) and indirect through anti-Jo-1 antibodies (following multiplicative rules: β = 0.42 × 0.41 = 0.17). The effect of CRP on CK was only indirectly mediated by BAFF and anti-Jo-1, but was significant (β = (0.52 × 0.29) + (0.52 × 0.42 × 0.41) = 0.24; *p* = 0.001). Modification of this model for subgroups of patients with DM and PM also supported direct effects of BAFF and anti-Jo-1 antibodies on CK and of CRP to BAFF (data not shown).

Alternative models testing whether CRP levels could result from the muscular or lung activity did not exhibit acceptable fit.

### Time changes in serum levels of BAFF, anti-Jo-1 antibodies, and disease activity, their mutual associations, and effect of treatment with glucocorticoids

A time-related decrease in disease activity and BAFF levels was documented by a negative correlation of time elapsed from diagnosis until sampling with laboratory markers of muscle involvement (CK, myoglobin, LDH, ALT, and AST; *r* = −0.32, −0.32, −0.30, −0.40, and −0.32, respectively; *p* < 0.05 for all; *n* = 63, 53, 55, 62, and 61, respectively) and clinical disease activity (cutaneous, skeletal, pulmonary, extramuscular global, muscle, physician’s global disease activity and MYOACT Score; *r* = −0.34, −0.36, −0.41, −0.62, −0.40, −0.65 and, −0.55 respectively; *p* < 0.01 and *n* = 58 for all). The negative association of serum levels of BAFF (*r* = −0.55, *p* = 0.002, *n* = 30) as well as marginally significant negative association of anti-Jo-1 levels (*r* = −0.35, *p* = 0.06, *n* = 30) with disease duration was documented in early patients.

Between the first two samplings, a significant reduction in myoglobin (median from 94 to 62.8 μg/l; *p* = 0.001) and a borderline significant drop in CK (from 2.62 to 1.22 μcat/l; *p* = 0.07) in the total group was recorded, as well as a significant decrease in other muscle enzymes (LDH, ALT, and AST; *p* < 0.05) in the early patient group. The clinical activity decreased significantly (*p* < 0.05 for all) in cutaneous, pulmonary, global extraskeletal muscle, muscle, and physician’s global disease activity between assessments at the first (VAS medians 1, 12.5, 15, 11, and 17.5 mm) and second (0, 4, 5, 6, and 11 mm) time points.

The significant reduction in BAFF levels between the two time points was documented within the entire patient group (from 1.8 to 1.3 ng/ml; *p* = 0.04) and was more pronounced in early patients (from 1.6 to 1.05 ng/ml; *p* = 0.02). In total, serum BAFF levels decreased to below the cut-off in 37.5% of patients with high BAFF, but an increase above the cut-off was recorded in 27% of patients with initially low BAFF levels. This non-linear variability of BAFF levels over time was also seen in a growth model as described below.

No significant change in anti-Jo-1 antibody levels between two time points was found (from 153.9 to 102.8 kU/l, *p* = 0.93). The serum levels of anti-Jo-1 antibodies detected in the first and second samplings were closely correlated (*r* = 0.70, *p* < 0.0001, *n* = 46), which corresponds to lower variability of anti-Jo-1 with time.

We further analysed correlations between changes in serum levels of BAFF or anti-Jo-1 and changes in laboratory markers and clinical disease activity in individual patients (Δ = value at first visit minus value at second visit). The changes in BAFF were associated with ΔCK, Δmyoglobin, ΔALT, and ΔAST, and showed a trend to association with Δcutaneous, Δmuscle, Δglobal, and Δskeletal disease activities (Table 3 and Additional file 5, left panel). The Δanti-Jo-1 antibody levels correlated only with the change in laboratory markers ΔAST and marginally with ΔALT, Δmyoglobin, and ΔCRP (Table 3 and Additional file 5, centre panel).

**Table 3:** Correlations of changes between first two visits (Δ = first visit – second visit) in levels of BAFF or anti-Jo-1 antibodies with change of disease activity and their association with prior daily dose of glucocorticoids given at initial assessment

	Change in BAFF			Change in anti-Jo-1			GC daily dose		
	n	r	*p*	*n*	*r*	p	*n*	*r*	*p*
Δ BAFF	–	–	–	44	0.18	0.25	43	0.31	0.04^a^
Δ CK	44	0.75	< 0.0001***	42	0.24	0.13	41	0.23	0.14
Δ Myoglobin	36	0.64	< 0.0001***	34	0.44	0.009	33	0.35	0.04
Δ ALT	45	0.52	0.0003**	43	0.31	0.04	42	0.33	0.03
Δ AST	43	0.63	< 0.0001***	41	0.54	0.0003**	40	0.30	0.05
Δ CRP	42	0.32	0.04	40	0.33	0.04	39	0.27	0.10
Δ Muscle VAS	39	0.33	0.04	37	−0.12	0.49	39	0.28	0.08
Δ Global VAS	39	0.38	0.02	37	−0.05	0.78	39	0.38	0.02
Δ Skeletal VAS	39	0.45	0.004**	37	0.1	0.56	39	0.06	0.72
Δ Cutaneous VAS^b^	12	0.64	0.03^a^	11	−0.03	0.92	12	0.38	0.22
Δ Pulmonary VAS^c^	30	−0.01	0.94	28	−0.30	0.12	30	0.42	0.02^a^

*ALT* alanine aminotransferase, *AST* aspartate aminotransferase, *BAFF* B-cell activating factor of the tumour necrosis factor family, *CK* creatine kinase, *CRP* C-reactive protein, *GC* glucocorticoids, *VAS* visual analogue scale

*p* = *p* value; **alfa = 0.01 (0.001 after Bonferroni correction); ***alfa = 0.001 (0.0001 after Bonferroni correction).

^a^No multiple testing correction applicable

^b^Evaluated within patients with dermatomyositis

^c^Evaluated within patients with interstitial lung disease

Changes in BAFF were associated with the daily dose of GC used at the time of first blood sampling. The change in disease activity assessed by Δmyoglobin, ΔALT, ΔAST, Δpulmonary, and Δglobal VAS also reflected the effect of GC therapy (Table 3, right column). Thus, higher doses of GC were followed by a larger decrease of serum levels of BAFF and with a decrease in some indicators of disease activity, but not with change in the anti-Jo-1 levels (*r* = 0.10, *p* = 0.54).

Finally, the changes in levels of anti-Jo-1 and BAFF were not mutually significantly associated, with a trend to improve after the exclusion of a single outlying value of Δanti-Jo-1 (Additional file 5).

The proportion of patients with high serum CRP decreased from an initial 38% to 28% at the second sampling, but no significant quantitative decrease in CRP levels was recorded. However, the changes in CRP were associated with changes in BAFF and anti-Jo-1 (Table 3), Δmyoglobin (*r* = 0.33, *p* < 0.05, *n* = 36) and weakly with Δconstitutional disease activity (*r* = 0.31, *p* = 0.07, *n* = 36).

### Longitudinal relations between serum levels of BAFF or anti-Jo-1 antibodies and disease activity markers or CRP

**Fig. 2:**
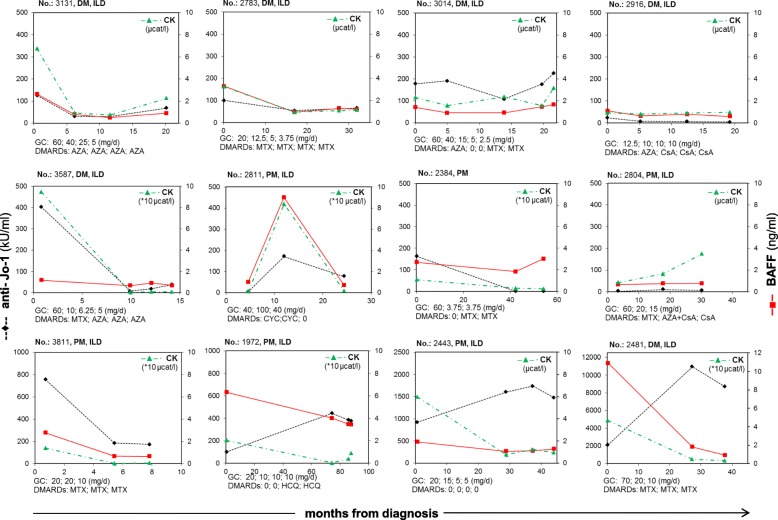
The time variability monitoring of B-cell activating factor of the tumour necrosis factor family (BAFF), anti-Jo-1-antibodies, and creatine kinase (CK) levels in serum of patients with myositis (dermatomyositis (DM) = 6; polymyositis (PM) = 6) longitudinally followed from an early period of disease (1.0 ± 1.4 months after diagnosis determination) receiving therapy. The dosage of glucocorticoids (GC) or treatment with disease-modifying anti-rheumatic drugs (DMARDs) from respective time points of sampling is listed under each graphical window. All patients except one had interstitial lung disease (ILD) as expressed in the graphical titles. The serum levels of BAFF (plain red lines) show similar course within time with CK (dash-and-dotted green lines) as well as with anti-Jo-1 (broken black lines) in the majority of cases. Left ordinate shows the anti-Jo-1 autoantibody levels in serum and right ordinate shows the serum levels of BAFF or CK. The abscissae show time intervals in months. MTX methotrexate; AZA azathioprine, CsA cyclosporine A, CYC cyclophosphamide, HCQ hydroxychloroquine

The time variability in serum levels of BAFF, anti-Jo-1, and CK showed comparable trends within time in a majority of patients who were followed from the early period after the diagnosis (illustrated in Fig. 2 with diagrams of 12 patients who had samples from three and more time points and short disease duration at first sampling; 1.0 ± 1.4 months after diagnosis determination). In general, high levels of serum BAFF declined after initial treatment with a high dose of GC, tended to rise in individual cases after GC tapering, and were parallel to CK levels more closely than anti-Jo-1 antibody levels (Fig. 2).

Anti-Jo-1 antibody levels became negative during follow-up in seven out of 40 patients (17.5%) within 0.5–4.3 years, in association with a significant decrease in serum levels of BAFF (median from 1.9 to 0.7 ng/ml; *p* = 0.02) and a decrease in disease activity measured as CK, myoglobin, ALT, and AST (*p* < 0.05). These patients were treated with a combination of GC (median daily dose 27.5 mg) and DMARDs (3 × azathioprine, 2 × methotrexate, 1 × cyclosporine A, and 1 × cyclophosphamide).

At a group level, statistical analysis of repeated measures with HLM showed high intra-individual variability in serum levels of BAFF over time (Table 4). This suggests that the majority (97%) of the variability of BAFF levels depends on time-related changes within the individual patient (ICC = 0.03), whereas 68% of the variability of anti-Jo-1 levels was explained by inter-individual differences between patients (ICC = 0.678).

**Table 4:** Hierarchical linear model (HLM) of long-term relations of serum levels of BAFF and anti-Jo-1 antibodies with serum levels of CK, CRP, and disease activity

Predictors	ICC^a^	Explained variance (%)^b^			Intercept						Slope^c^					
		BAFF	anti-Jo-1	CK	BAFF (ng/ml)	*p*	anti- Jo-1 (kU/l)	*p*	CK (μkat/l)	*p*	BAFF (ng/ml)	*p*	anti- Jo-1 (kU/l)	*p*	CK (μkat/l)	*p*
Time from diagnosis^d,e^	–	0	62.3	31.2	1.85	˂ 0.001	340.9	0.04	9.06	0.04	0.01	0.10	11.9	0.21	−0.06	0.63
BAFF^e^ (ng/ml)	0.030	–	79.1	49.6	–	–	748.0	0.03	0	0.99	–	–	91.6	0.22	4.34	0.003
anti-Jo-1^e^ (kU/l)	0.678	31.6	–	72.3	2.33	˂ 0.001	–	–	1.4	0.34	0.002	0.03	–	–	0.02	0.05
CK^e^ (μkat/l)	0.100	75.6	73.8	–	2.30	˂ 0.001	747.1	0.03	–	–	0.14	0.02	7.14	0.59	–	–
CRP^f^ (mg/l)	0.107	74.3	70.2	44.1	2.35	˂ 0.001	749.5	0.03	8.7	0.02	0.15	0.08	− 21.7	0.76	0.98	0.07
Pulmonary VAS^g^ (mm)	0.582	61.7	60.4	ND	2.32	˂ 0.001	785.8	0.03	ND	–	0.11	0.16	−16.5	0.03	ND	–
Muscle VAS^g^ (mm)	0.319	84.5	71.9	ND	2.32	˂ 0.001	784.4	0.03	ND	–	−0.12	0.54	−7.8	0.22	ND	–
Global VAS^g^ (mm)	0.241	28.3	71.2	ND	1.30	˂ 0.001	939.3	0.07	ND	–	0.06	0.01	−7.4	0.21	ND	–

Dependent variables are situated in columns and predictors in rows

*BAFF* B-cell activating factor of the tumour necrosis factor family, *CK* creatine kinase, *CRP* C-reactive protein, *ICC* intra-class correlation coefficient, *ND* not done, *VAS* visual analogue scale

^a^ICC; the ratio of the between-individuals variance (level 2) to the total variance

^b^% of variance explained at level 1 (within individuals): pseudo *R*^2^; based on HLM

^c^Average effect of explanatory variables (Slope) was estimated using maximum likelihood method with robust standard errors

^d^Average change over time (months from diagnosis); based on the unconditional growth model

^e^Determined in 80 repeated measures on 23 patients

^f^Determined in 79 repeated measures on 23 patients

^g^Determined in 73 repeated measures on 22 patients

The percentage of explained variance within patients in the unconditional growth model with time from diagnosis as an independent variable (Table 4) showed that linear regression is a good model for anti-Jo-1 (62%) and marginally acceptable for CK (31%), but not appropriate for BAFF (0%) levels in serum. BAFF was a good predictor of within-patient variability of anti-Jo-1 (it explained 79% of anti-Jo-1 variance) but the opposite prediction was not found, which corresponds to the dependence of anti-Jo-1 levels on BAFF detected by the PA model as described above (Fig. 1b).

The close longitudinal associations between serum levels of BAFF or anti-Jo-1 antibodies with markers of muscle involvement and clinical disease activity were supported, with a high proportion of their variance explained by CK, CRP, pulmonary, and muscle VAS (BAFF: 76%, 74%, 61%, and 85%; or anti-Jo-1: 74%, 70%, 60%, and 72%). Moreover, anti-Jo-1 antibody levels could be predicted with global disease activity (71%).

The extended interpretation of the further results of HLM is provided in detail in Additional file 2.

## Discussion

The main finding of this study was a strong correlation between serum levels of BAFF and anti-Jo-1 antibodies and their association with disease activity variables and clinical phenotype of myositis-associated ILD. In addition, we characterised differences in time-related variability of BAFF and anti-Jo-1 serum levels during longitudinal follow-up. Whereas serum levels of BAFF were associated with treatment within the same individual, the levels of anti-Jo-1 were more stable and did not reflect currently used therapy. Finally, we recorded associations of the serum levels of CRP with BAFF, anti-Jo-1, and markers of muscle impairment.

Higher serum levels of BAFF compared with healthy individuals confirm previous reports in unselected cohorts of patients with myositis [17, 33–35]. Here, we analysed the whole accessible cohort of anti-Jo-1-positive patients with myositis. The higher proportion of PM patients compared with DM is in accordance with the known higher frequency of anti-Jo-1 in PM [36], and the high proportion of patients with ILD compared with the overall myositis population [37] corresponds with the strong association of ILD with anti-Jo-1 positivity [19, 38]. The numerically more frequent ILD in patients with PM compared with those with DM is in agreement with already published data and, again, can be explained by the anti-Jo-1 status [39].

Contrary to earlier reports in autoantibody heterogeneous [17] or not defined [34] cohorts of myositis patients, we did not find a difference in serum levels of BAFF between patients with DM and PM subgroups or higher BAFF levels in patients with ILD within the current autoantibody homogenous, anti-Jo-1-positive cohort. However, the present findings confirm our previous results of multiple regression analysis showing that diagnosis of PM or DM was less discriminatory for BAFF levels than the presence of anti-Jo-1 antibodies or lung involvement, and explaining higher serum BAFF levels in myositis-associated ILD by the presence of anti-Jo-1 antibodies [17]. We also found a close correlation between serum levels of BAFF and anti-Jo-1, particularly in early cases and in patients with PM or in PM/DM patients with ILD. A similar association between serum levels of BAFF and other disease-specific autoantibodies has been reported, for example anti-dsDNA or anti-Smith antibodies in SLE [9, 10, 13, 16, 22, 40, 41], rheumatoid factor (RF) or anti-CCP antibodies in rheumatoid arthritis (RA) [10, 42], and RF or anti-SSA/B antibodies in Sjögren’s syndrome [14], suggesting a close relationship between BAFF and autoantibody production. A biologic association between BAFF and anti-Jo-1 antibody levels was supported by our path analysis which revealed a dependence of anti-Jo-1 levels on BAFF levels in serum. A possible causal relationship was further corroborated by longitudinal analysis with the HLM, where a high proportion of anti-Jo-1 variance was explained by serum levels of BAFF, but the converse was not true. This analysis, together with the reported quantitative association of BAFF with another MSA anti-MDA5 (melanoma differentiation-associated gene 5) in juvenile DM [35], further supports involvement of a disease-specific inflammatory process in the production of autoantibodies triggered by BAFF release into the circulation in inflammatory myopathies.

Studies from other systemic diseases (SLE and RA) reported limited variation of serum levels of BAFF over time and no relation to disease flares [9, 13]. However, our findings of changes in serum levels of BAFF over time in association with changes in disease activity was similar to a longitudinal follow-up in systemic sclerosis (SSc), early RA, and SLE [8, 41, 42], indicating that BAFF may have a higher impact in early disease.

Only 54% of our anti-Jo-1-positive patients had elevated baseline serum levels of BAFF, and the levels varied considerably in association with the laboratory markers of muscle impairment, which confirms previous reports [17, 34]. These correlations were expressed particularly early after the diagnosis and in subgroups of patients with PM or ILD, further supporting the hypothesis that BAFF is more involved in early phases of this disease and that other factors may also influence autoantibody production.

Moreover, serum levels of BAFF were associated with cutaneous disease activity in DM patients. An association of serum BAFF levels with severity of skin sclerosis in SSc, with higher numbers of plaque lesions in localised scleroderma and with malar rash in SLE [8, 12, 40], has been reported previously. All these associations point to a possibility that active skin lesions might be a source of BAFF.

The main variability of serum levels of BAFF was seen in time-related changes within individual patients and a substantial number of patients expressed reduction from high to low levels between two time points, but the opposite, an increase in BAFF levels, also occurred. These changes correlated with changes in markers of muscle impairment and also with changes in cutaneous, muscular, skeletal, and global disease activity. The close longitudinal relationship between disease activity and serum BAFF levels was further supported by a high proportion of its variance being explained by serum CK levels and muscle or pulmonary VAS in analysis with HLM. This, together with the position of BAFF in the PA model, may suggest BAFF as a possible direct mediator of disease mechanisms in patients with anti-Jo-1-positive myositis.

The changes between two samplings in serum levels of BAFF and laboratory and clinical disease activity measures showed a trend towards an association with initial daily glucocorticoid doses. Similar associations were also seen in patients with SLE or SSc [9, 12]. We have previously reported an association of an early decrease of BAFF levels with cumulative glucocorticoid doses at the beginning of treatment in early myositis cases [17], and our current findings provide further support for the sensitivity of BAFF production to glucocorticoid treatment in myositis.

The anti-Jo-1 antibody became negative in patients with a decrease of disease activity (in 18%), which was preceded by intensive treatment and associated with a decrease in serum BAFF levels. This is similar to previous reports of the disappearance of anti Jo-1 autoantibodies in periods of disease inactivity [18, 43, 44]. This could also be an explanation for the low anti-Jo-1 antibody levels in six already treated patients who had positive anti-Jo-1 levels in sera taken before the study start.

Similar to BAFF, the serum levels of anti-Jo-1 antibodies correlated with serum markers of muscle impairment. The results of our unconditional growth model demonstrated associations between variations in anti-Jo-1 antibody levels over time and with muscular, pulmonary, and global disease activity comparable to longitudinal observations analysed with mixed linear regression for repeated measures reported by Stone et al. [18], and supports the role of anti-Jo-1 in disease mechanisms.

An unexpected finding in our study was the association of serum levels of CRP with levels of BAFF and anti-Jo-1 antibodies. Besides the association with change of constitutional activity, CRP also reflected muscle impairment by correlation with CK and myoglobin in serum both at baseline and longitudinal follow-up. These associations were recorded despite the modest elevations in CRP levels. Although CRP is not considered to be a marker of disease activity in most systemic diseases, some studies indicates its role mainly in patients with lung involvement [45], including myositis [19–21, 46]. We recorded more pronounced associations of muscle impairment markers with CRP in a subset of patients with ILD, but no correlation of CRP levels in serum with pulmonary activity. In our PA model, CRP displayed significant effects on serum levels of BAFF, similar to that reported in SLE (Fig. 1b) [13, 22, 47]. This finding could be explained by the described capability of CRP to trigger the release of soluble, biologically active BAFF from myeloid cells by FcγΙa receptor engagement [48]. These mechanisms, however, need further exploration.

A limitation of our study is the wide heterogeneity of disease activity with low median values (Table 1), which may explain the absence of associations between serum levels of BAFF and clinical activity measures or anti-Jo-1 at baseline, as seen by other authors [18, 34]. The low disease activity in some patients could possibly be explained by the intensive treatment that patients received before inclusion in our study.

## Conclusion

In summary, levels of anti-Jo-1 in serum are proposed as a biomarker of disease activity in patients with anti-Jo-1-positive myositis. Our study demonstrated that serum levels of BAFF correlate with serum levels of anti-Jo-1 antibodies. Moreover, the longitudinal analysis showed a high intra-individual variability of serum levels of BAFF over time which could predict 79% of anti-Jo-1 level variance, whereas the anti-Jo-1 variability was explained by inter-individual differences. A relationship between serum levels of BAFF, anti-Jo-1, and disease activity was supported by high proportions of their variance being explained by serum levels of CK and CRP or pulmonary and muscle activities. The finding of an association between serum levels of CRP and levels of BAFF, anti-Jo-1 antibodies, or markers of muscle impairment was surprising, although consistent with some reports in other systemic diseases.

Associations between serum levels of BAFF and anti-Jo-1 antibodies with disease activity measures support a possible role of BAFF in the pathogenesis of anti-Jo-1-positive myositis. The anti-Jo-1 autoantibody was recently described as one of the strongest predictors of response in rituximab-treated myositis patients in the RIM trial [49]. Our finding of an association of anti-Jo-1 levels with BAFF, particularly in patients with PM and ILD, also implies that BAFF-blocking therapy could be an attractive novel treatment for these patients.

## Additional files

Additional file 1: Design of data collection. Graphical representation of the time flow of individual patient visits. The blue background highlights cases followed for more than two time points. (PDF 122 kb)

Additional file 2: The application method of the hierarchical linear model (HLM). Detailed explanation of HLM principles, its use for the unbalanced designs, and interpretation of results. (PDF 199 kb)

Additional file 3: The scatter plots of source cross-sectional data for correlational analysis presented in Table 2. The values of levels of BAFF, anti-Jo-1 antibodies, and CRP in serum are plotted in columns against the serum levels of markers of muscle impairment (CK, myoglobin, and AST) and CRP in rows. Based on the non-normal distribution, the logarithmically transformed data are plotted. Statistics are: *r* = Spearman’s correlation coefficient; p = *p* value. (PDF 540 kb)

Additional file 4: Multivariate analysis of the cross-sectional relationships between serum levels of BAFF, anti-Jo-1 antibodies, CK, and CRP. The detailed explanation of modifications of the multiple regression model and its variants accomplished in the path analysis and principles of interpretation. (PDF 193 kb)

Additional file 5: The scatter plots of source data for correlational analysis presented in Table 3. Changes between first two visits (Δ = 1st visit – 2nd visit) of (A) BAFF plotted against Δanti-Jo-1, changes in both BAFF and anti-Jo-1 plotted in columns against parameters of activity in rows. These are: (B) changes in markers of muscle impairment (ΔCK, Δmyoglobin, ΔALT and ΔAST) and (C) changes in clinical disease activity assessments (Δmuscle, Δglobal, Δskeletal within the entire patient group, Δcutaneous within patients with dermatomyositis (DM), and Δpulmonary within patients with lung involvement (ILD)). Statistics are: *r* = Spearman’s correlation coefficient; p = *p* value. A single outlying value of Δanti-Jo-1 is highlighted by a red circle. The graphs with exclusion of the outlier are plotted in the right column. The significance of some correlations became even stronger after exclusion of the outlier. (PDF 535 kb)

## Abbreviations

ALT: Alanine aminotransferase
ANOVA: Analysis of variance
AST: Aspartate aminotransferase
BAFF: B-cell activating factor of the tumour necrosis factor family
CFI: Comparative fit index
CK: Creatine kinase
CRP: C-reactive protein
DM: Dermatomyositis
DMARD: Disease-modifying anti-rheumatic drug
ELISA: Enzyme-linked immunosorbent assay
GC: Glucocorticoids
HLM: Hierarchical linear model
ICC: Intra-class correlation coefficient
ILD: Interstitial lung disease
IMACS: International Myositis Assessment and Clinical Studies
MDAAT: Myositis Disease Activity Assessment Tool
MR: Multiple regression
MSA: Myositis-specific antibodies
PA: Path analysis
PM: Polymyositis
PNFI: Parsimony normed fit index
RA: Rheumatoid arthritis
RF: Rheumatoid factor
RMSEA: Root mean square error of approximation
SLE: Systemic lupus erythematosus
SSc: Systemic sclerosis
VAS: Visual analogue scale
