# Supplementary material for: Serum levels of B-cell activating factor of the TNF family (BAFF) correlate with anti-Jo-1 autoantibodies levels and disease activity in patients with anti-Jo-1positive polymyositis and dermatomyositis
Source: Arthritis Res Ther. 2018 Jul 27;20:158. doi: 10.1186/s13075-018-1650-8 (PMC6062864; doi:10.1186/s13075-018-1650-8)

Additional Figure 1.: Unbalanced design of data collection. Samples analyzed with HLM are highlighted by background.

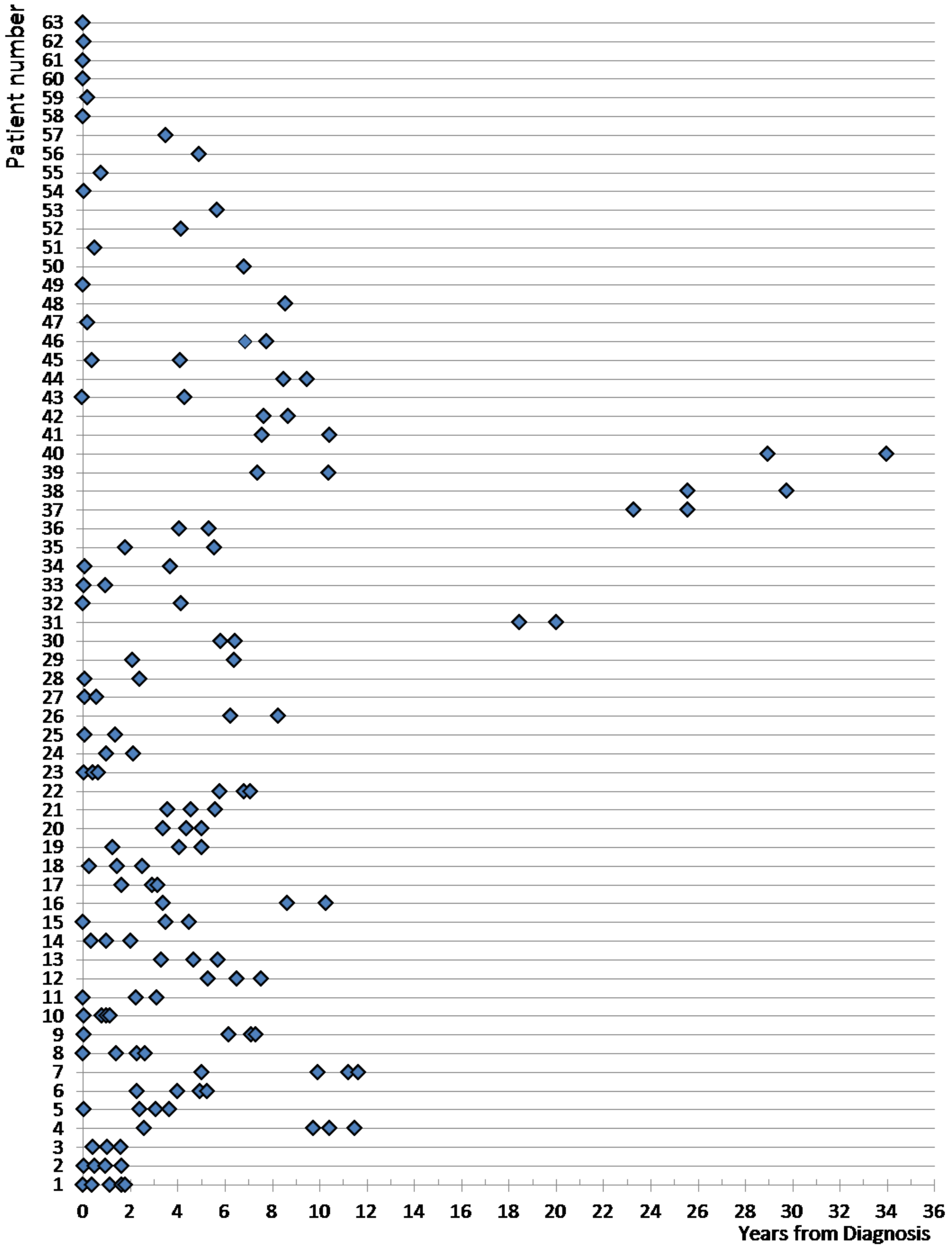

Supplement: Supplementary file 1 — Design of data collection. Graphical representation of the time flow of individual patient visits. The blue background highlights cases followed for more than two time points. (PDF 122 kb) [file 13075_2018_1650_MOESM1_ESM.pdf]
