# Supplementary material for: Serum levels of B-cell activating factor of the TNF family (BAFF) correlate with anti-Jo-1 autoantibodies levels and disease activity in patients with anti-Jo-1positive polymyositis and dermatomyositis
Source: Arthritis Res Ther. 2018 Jul 27;20:158. doi: 10.1186/s13075-018-1650-8 (PMC6062864; doi:10.1186/s13075-018-1650-8)
Supplement: Supplementary file 2 — The application method of the hierarchical linear model (HLM). Detailed explanation of HLM principles, its use for the unbalanced designs, and interpretation of results. (PDF 199 kb) [file 13075_2018_1650_MOESM2_ESM.pdf]

The application of the hierarchical linear model (HLM)

Method

For evaluation of long term relations between BAFF and anti-Jo-1 levels in serum and activity assessment, data from 26 patients who were tested in 3 and more time points were analyzed. A two-level hierarchical linear model (HLM) was used here, because the present data set is partitioned into two levels:

- The lower level (Level-1) – repeated time point measures (3 and more occasions), evaluating variability within individuals.
- The higher level (Level-2) – patients (n=26), evaluating variability between individuals.

Two dependent variables, BAFF and anti-Jo-1, are measured at the lower level. A total of 10 independent variables are situated also at the lower level, because they are measured repeatedly in time at the same occasions as the dependent variables. Let us note that HLM was designed for BAFF and anti-Jo-1 separately. The analytic procedure of multilevel analysis with longitudinal data involves several steps.

The very first model is the *unconditional model*, which should always be performed [1]. This model includes no independent variables at any level. The unconditional model allows us to evaluate the relative magnitude of the within-patients (level-1) and between-patients (level-2) variance components. Their proportion is expressed by the intraclass correlation coefficient (ICC), which describes the proportion of the total variance of BAFF and/or anti-Jo-1 that lies between patients.

The second step is the *unconditional growth model*, in which the time is the only independent variable. The main purpose of this step is to reveal possible linear trend over time. Each patient has his/her unique regression line fitted to the repeated measures, which leads to the estimates of 26 different regression coefficients, intercepts and slopes. These coefficients are subsequently allowed to vary randomly across patients. Special attention was paid to the coding of the time variable. The time at first measurement occasion is equal to the time elapsed from diagnosis and date of diagnosis within each patient was set to zero. Thus, all patients had a unique set of 3 and more measurements with unequal distance between time points. For better illustration, the time flow of individual visits is represented graphically in the Supplementary Figure 1 (Additional file 1). This type of unbalanced design doesn't allow using traditional methods for analysis of longitudinal panel data like repeated measures ANOVA or MANOVA. Mixed linear regression analysis for repeated measures was used to handle the problem with unbalanced longitudinal data in a previous study [1]. Let us note that

this model belongs to the family of equivalent statistical models known under a variety of names including HLM, multilevel analysis, two-stage models, random-effect models, variance component models, random coefficient models, random regression models, and/or empirical Bayes models [2].

The third step is to incorporate all remaining time-varying independent variables. Due to the small number of repeated measures, only *one independent and one dependent variable* were analyzed at level-1. The percentage of the variance of either BAFF, anti-Jo-1 or CK explained by the particular time-varying variable (pseudo R^2) was used as an index of fit. The fixed parts of the model (intercepts and slopes) were estimated using maximum likelihood method with robust standard errors.

All analysis of longitudinal data has been conducted in HLM 6 [3], a specialized package for HLM.

Results:

The results of HLM analysis are presented in Table 4. The ICCs of all time-varying variables regardless dependent or independent are situated in the second column of Table 4. The highest value of the ICC was observed in the case of anti-Jo-1 (0.678) which indicates that about 68% of anti-Jo-1 variability is situated between patients (level-2). By contrast, the lowest value of ICC was found in BAFF (0.030) which means that only about 3% of the total variance is situated between patients and remaining 97% is due to the within-patient variability in time. The values of ICCs are neither too low nor too high and, thus, give us some support for accomplishing consequential steps, because there is (on average) substantial amount of variables' variability at both levels.

The results of the unconditional growth model are represented by the first row in Table 4. Time from diagnosis in months is the only independent variable here. The degree of how appropriate the linear function is to fit the time points within patients is expressed by the percentage of explained variance at level-1. By inspecting the % of the variance explained by the time from diagnosis variable, we may conclude that linear regression is a good model for anti-Jo-1 (62.3%) and marginally acceptable for CK (31.2%), but not appropriate for BAFF (0.0%) within patients. In the case of anti-Jo-1 there is a linear change in time within patients, nevertheless, the average slope (11.9) is not significant due to the large variability of slopes within individuals.

Subsequently, the time from diagnosis variable was replaced by remaining 7 predictors. The findings indicate that CK, Muscle VAS and CRP are the best predictors of both anti-Jo-1 and BAFF. All variables explained more than 70% of the variability at the level-1. Moreover,

the unequal magnitude of the percentage of variance explained between anti-Jo-1 and BAFF was caused by different number of data points that satisfy HLM requirements and assumptions on dependent variables. Therefore, it seems to be more reasonable to predict anti-Jo-1 from BAFF (with 79.1% of explained variance) than conversely (31.6%). BAFF is also important predictor of CK (49.6%), which can be well estimated by anti-Jo-1 (72.3%). It is obvious that the mutual longitudinal relationships between anti-Jo-1 and all other variables are very high. On the other hand, BAFF cannot be easily estimated by other variables except CRP (74.3%), CK (75.6%), and partially Pulmonary VAS (61.7%). The very last six columns in Table 4 describe to which extent the regression lines are parallel. If the p-values of the slopes are significant, there is a little variability of slopes within patients. In the case of anti-Jo-1, only slope of the Pulmonary VAS was significant (p=0.03). The negative value of the slope (-16.5) means that, on average, the higher value of Pulmonary VAS the lower value of anti-Jo-1. The slopes of all other predictors of anti-Jo-1 were not significantly different from zero. In the event of BAFF, the slope of three time-varying variables are significant at 5% level and one at 10% level, but with regard to % of the variance explained only CK (p=0.02) and CRP (p=0.08) can be considered as a good predictors. The estimates of slopes of CK (0.14) and CRP (0.15) indicate strong positive longitudinal relationships between these two variables and serum BAFF levels.

References:

1. Singer JD, Willett JB: **Applied longitudinal data analysis: Modeling change and event occurrence** *MA: New York: Oxford University Press* 2003:45-74
2. Hedeker D, Gibbons R: **Longitudinal Data Analysis**. *New Jersey: Wiley* 2006:47.
3. Raudenbush SW, Bryk AS, Cheong YF, Congon R, duToit M: **HLM 6: Hierarchical linear and nonlinear modeling**. *MA: Lincolnwood, IL: Scientific Software International, Inc* 2004:14-64.
