# Supplementary material for: Serum levels of B-cell activating factor of the TNF family (BAFF) correlate with anti-Jo-1 autoantibodies levels and disease activity in patients with anti-Jo-1positive polymyositis and dermatomyositis
Source: Arthritis Res Ther. 2018 Jul 27;20:158. doi: 10.1186/s13075-018-1650-8 (PMC6062864; doi:10.1186/s13075-018-1650-8)
Supplement: Supplementary file 3 — The scatter plots of source cross-sectional data for correlational analysis presented in Table 2. The values of levels of BAFF, anti-Jo-1 antibodies, and CRP in serum are plotted in columns against the serum levels of markers of muscle impairment (CK, myoglobin, and AST) and CRP in rows. Based on the non-normal distribution, the logarithmically transformed data are plotted. Statistics are: r = Spearman’s correlation coefficient; p = p value. (PDF 540 kb) [file 13075_2018_1650_MOESM3_ESM.pdf]

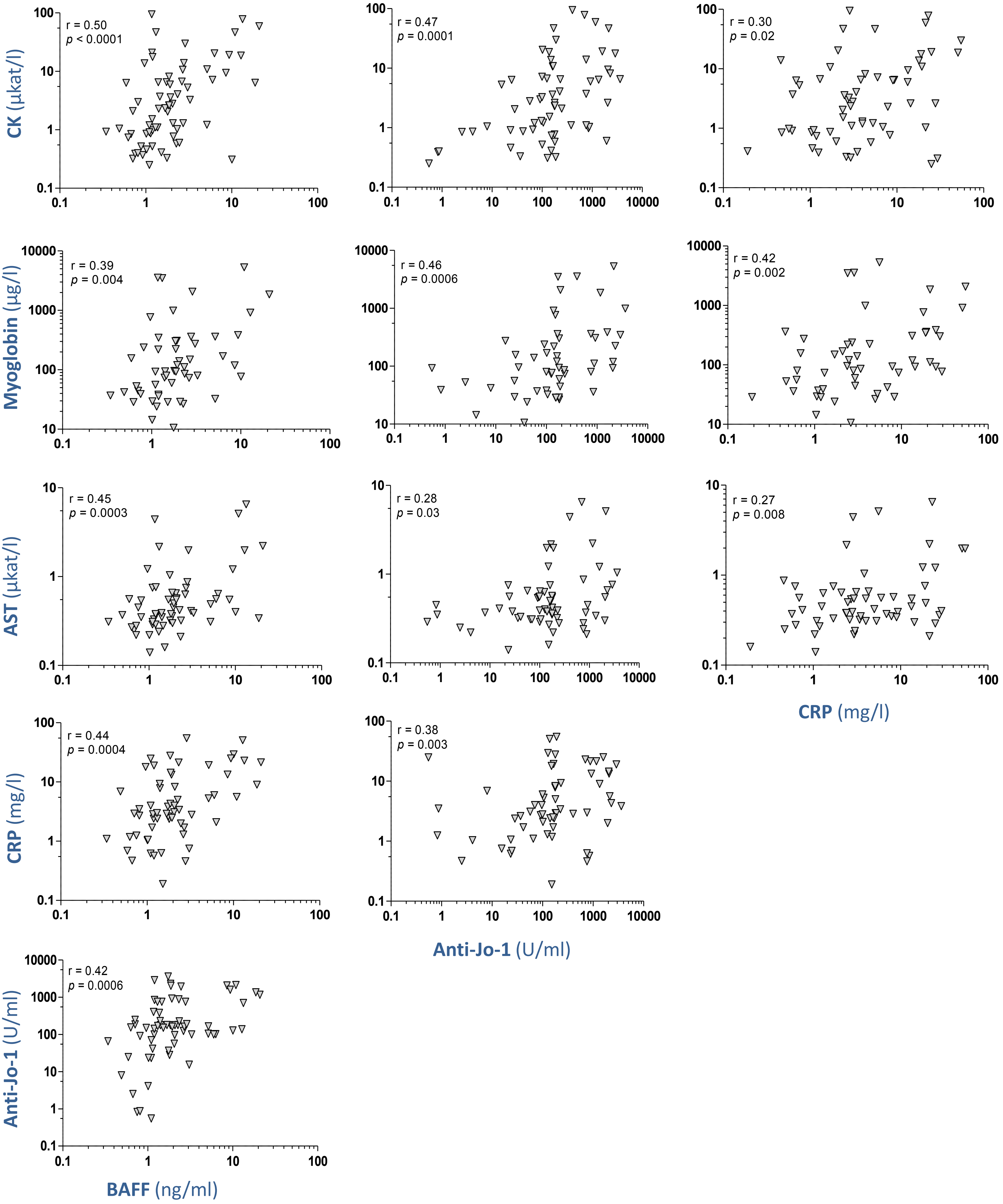

Additional file 4: The scatter plots of source cross-sectional data for correlational analysis presented in Table 2.

The values of levels of BAFF, anti-Jo-1 antibodies and CRP in serum are plotted in columns against the serum levels of markers of muscle impairment (CK, myoglobin and AST) and CRP in rows. Based on the non-normal distribution, the logarithmically transformed data are plotted. Statistics are: r = Spearman´s correlation coefficient; p = p-value
