# Supplementary material for: Serum levels of B-cell activating factor of the TNF family (BAFF) correlate with anti-Jo-1 autoantibodies levels and disease activity in patients with anti-Jo-1positive polymyositis and dermatomyositis
Source: Arthritis Res Ther. 2018 Jul 27;20:158. doi: 10.1186/s13075-018-1650-8 (PMC6062864; doi:10.1186/s13075-018-1650-8)
Supplement: Supplementary file 4 — Multivariate analysis of the cross-sectional relationships between serum levels of BAFF, anti-Jo-1 antibodies, CK, and CRP. The detailed explanation of modifications of the multiple regression model and its variants accomplished in the path analysis and principles of interpretation. (PDF 193 kb) [file 13075_2018_1650_MOESM4_ESM.pdf]

Multivariate analysis of the cross-sectional relationships between serum levels of BAFF, anti-Jo-1 antibodies, CK and CRP.

The relationships among serum levels of BAFF, anti-Jo-1 antibodies and CRP (independent variables) and their possible influence on the muscle involvement represented by the serum levels of CK (dependent variable) was analysed by multiple regressions (MR).

Variables Entered/Removed

Model	Variables Entered	Variables Removed	Method
1	JO1, CRP, BAFF[a]	.	Enter

a. All requested variables entered.

Model Summary

Model	R	R Square	Adjusted R Square	Std. Error of the Estimate
1	,600[a]	,360	,327	,9416977

a. Predictors: (Constant), JO1, CRP, BAFF

ANOVA[b]

Model		Sum of Squares	df	Mean Square	F	Sig.
1	Regression	29,389	3	9,796	11,047	,000[a]
	Residual	52,321	59	,887		
	Total	81,710	62			

a. Predictors: (Constant), JO1, CRP, BAFF

b. Dependent Variable: CK

Coefficients[a]

Model		Unstandardized Coefficients		Standardized Coefficients	t	Sig.
		B	Std. Error	Beta		
1	(Constant)	,000	,119		,000	1,000
	CRP	,089	,141	,077	,627	,533
	BAFF	,656	,223	,377	2,938	,005
	JO1	,176	,072	,281	2,436	,018

a. Dependent Variable: CK

The results of MR, shown in the original output from SPSS above, are identical to the outcomes from LISREL when MR is considered as a special case of path analysis (PA) (Figure 1A). Both packages provide the same estimates of the standardized regression (path)

coefficients that are represented by the one-headed arrows in the path-diagram (Fig 1A). The two-headed arrows among the independent variables constitute bivariate correlations. The only difference between the LISREL and SPSS outputs consists in a residual variance. LISREL presents residual variance (Θ) not explained by the model (Θ = 0.64) whereas SPSS produces R square ($R^2 = 1 - 0.64 = 0.36$). According to MR conventions, CRP with non-significant path coefficient (β = 0.08; $P = 0.53$) would be excluded from further analysis using elimination procedure (e.g. stepwise), however, with respect to significant bivariate correlations of CRP with BAFF, anti-Jo-1 and CK it could still play a role in the whole system. From the PA point of view, MR is a saturated model (df = 0; chi-square = 0) and removing some paths (arrows) would result in an increase of both df and chi-square statistics. Moreover, PA allows to modify the default MR model by changing the type and direction of the arrows based on the theory-driven considerations.

In the current study, the modification of the MR model was performed as follows (Figure 1B): Mentioned non-significant path coefficient between CRP and anti-Jo-1 (β = 0.08) was fixed to zero (the arrow was removed) and the remaining correlations between independent variables (two-headed arrows) were replaced by the selected one headed arrows (introducing possible causal relationship). Next, all two-headed arrows (i.e., correlations) were replaced by their one-headed counterparts (i.e., path coefficients). As a consequence, the path coefficient between CRP and Jo-1 became non-significant and thus was also fixed to zero.

Generally speaking, variables not connected with an arrow exhibited non-significant direct effect; which, nevertheless, could be indirect. The effect of BAFF on CK was both direct and indirect (through anti-Jo-1 antibodies) (Figure 1B). Indirect effects follow multiplicative rules of path coefficients [e.g. total standardized indirect effect of CRP on CK: $\beta = (0.52 \times 0.29) + (0.52 \times 0.42 \times 0.41) = 0.24$; $P = 0.001$]. CRP is now again significant predictor of CK, but the effect is indirect and is mediated by BAFF and Jo-1.

The model depicted in figure 1B is nested within the model in figure 1A. Therefore, they can be compared using the chi-square difference test. By removing two arrows we increased df by 2 and chi-square from 0 to 1.24. The value of 1.24 with 2 degrees of freedom is not significant (P = 0.538). It means that the information loss was negligible and we obtained a simpler model with still acceptable fit (i.e., more parsimonious).

The further modification of this model for the subgroups of patients with DM or PM also supported direct effects of BAFF and anti-Jo-1 antibodies on CK and of CRP to BAFF (not shown).

Let us note that also alternative models with opposite direction of some arrows were tested. For example, to assess whether CRP levels could result from the local muscular or lung inflammatory activity, the model with an arrow from CK or Pulmonary VAS (Pulm) to CRP was examined. However, none of these opposite models exhibited acceptable fit.
