# Supplementary material for: Serum levels of B-cell activating factor of the TNF family (BAFF) correlate with anti-Jo-1 autoantibodies levels and disease activity in patients with anti-Jo-1positive polymyositis and dermatomyositis
Source: Arthritis Res Ther. 2018 Jul 27;20:158. doi: 10.1186/s13075-018-1650-8 (PMC6062864; doi:10.1186/s13075-018-1650-8)
Supplement: Supplementary file 5 — The scatter plots of source data for correlational analysis presented in Table 3. Changes between first two visits (Δ = 1st visit – 2nd visit) of (A) BAFF plotted against Δanti-Jo-1, changes in both BAFF and anti-Jo-1 plotted in columns against parameters of activity in rows. These are: (B) changes in markers of muscle impairment (ΔCK, Δmyoglobin, ΔALT and ΔAST) and (C) changes in clinical disease activity assessments (Δmuscle, Δglobal, Δskeletal within the entire patient group, Δcutaneous within patients with dermatomyositis (DM), and Δpulmonary within patients with lung involvement (ILD)). Statistics are: r = Spearman’s correlation coefficient; p = p value. A single outlying value of Δanti-Jo-1 is highlighted by a red circle. The graphs with exclusion of the outlier are plotted in the right column. The significance of some correlations became even stronger after exclusion of the outlier. (PDF 535 kb) [file 13075_2018_1650_MOESM5_ESM.pdf]

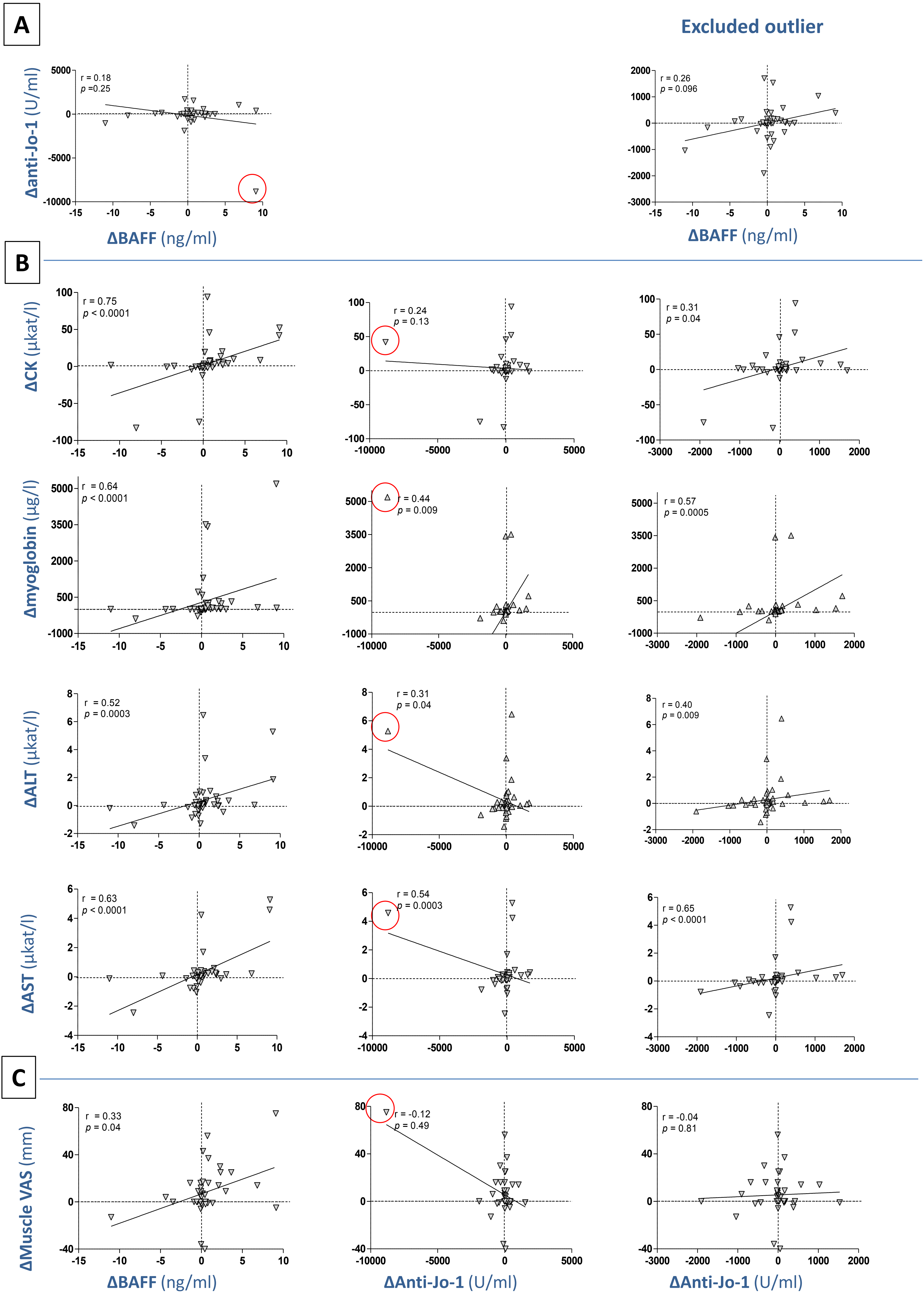
A
Excluded outlier
Δanti-Jo-1 (U/ml)
r = 0.18
p =0.25
ΔBAFF (ng/ml)
r = 0.26
p = 0.096
ΔBAFF (ng/ml)
B
ΔCK (µkat/l)
r = 0.75
p < 0.0001
r = 0.24
p = 0.13
r = 0.31
p = 0.04
Δmyoglobin (µg/l)
r = 0.64
p < 0.0001
r = 0.44
p = 0.009
r = 0.57
p = 0.0005
ΔALT (µkat/l)
r = 0.52
p = 0.0003
r = 0.31
p = 0.04
r = 0.40
p = 0.009
ΔAST (µkat/l)
r = 0.63
p < 0.0001
r = 0.54
p = 0.0003
r = 0.65
p < 0.0001
C
ΔMuscle VAS (mm)
r = 0.33
p = 0.04
r = -0.12
p = 0.49
r = -0.04
p = 0.81
ΔBAFF (ng/ml)
ΔAnti-Jo-1 (U/ml)
ΔAnti-Jo-1 (U/ml)

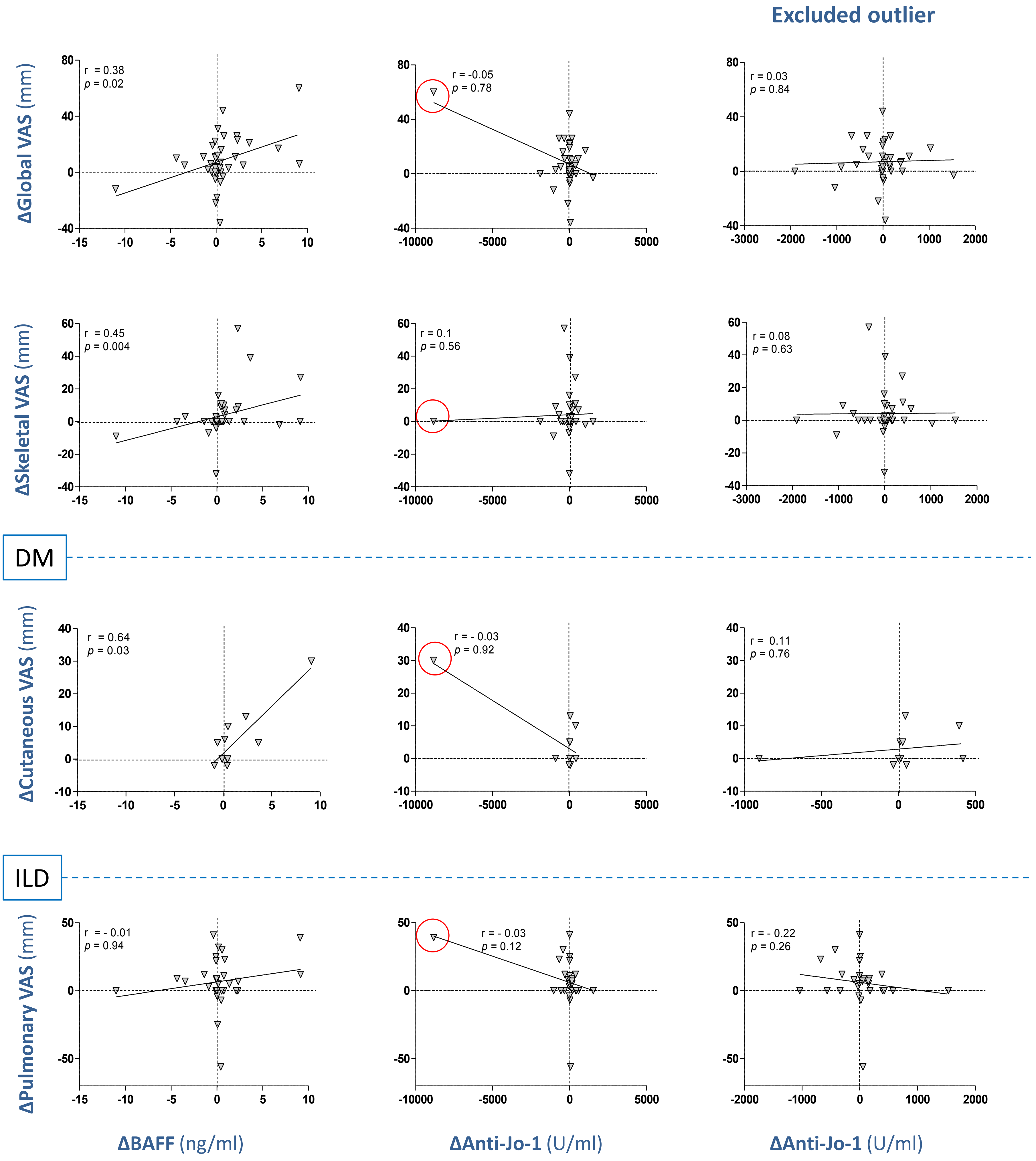

Additional file 5: The scatter plot of source data for correlational analysis presented in Table 3.

Changes between first two visits (Δ= 1st visit - 2nd visit) of (A) BAFF plotted against Δanti-Jo-1, changes of both BAFF and anti-Jo-1 plotted in columns against parameters od activity in rows. These are: (B) changes of markers of muscle impairment [ΔCK, Δmyoglobin, ΔALT and ΔAST)] and (C) changes of clinical disease activity assessments [Δmuscle, Δglobal, Δskeletal – within the entire patient´s group, Δcutaneous - within patient with dermatomyositis (DM) and Δpulmonary - within patients with lung involvement (ILD)]. Statistics are: r = Spearman´s correlation coefficient; p = p-value

A single outlying value of Δanti-Jo-1 is highlighted by red circle. The graphs with exclusion of the outlier are plotted in the right column. The significance of some correlations became even stronger after exclusion of the outlier.
